# Ultrasound features in early pregnancy for predicting abnormal karyotype in first‐trimester miscarriage

**DOI:** 10.1002/uog.70159

**Published:** 2026-01-03

**Authors:** T. Setty, S. L. Kastora, T. Tellum, J. Farren, E. Jauniaux, D. Jurkovic

**Affiliations:** ^1^ University College London, EGA Institute for Women's Health London UK; ^2^ Department of Gynecology Oslo University Hospital Oslo Norway; ^3^ Faculty of Medicine, Institute of Clinical Medicine University of Oslo Oslo Norway

**Keywords:** chromosomal aberration, crown–rump length, cytogenetic analysis, early diagnosis, first trimester, gestational sac, live pregnancy, maternal age, miscarriage, ultrasonography, yolk sac

## Abstract

**Objective:**

To investigate whether combining abnormal morphological features observed on ultrasound in live pregnancies that ended in a first‐trimester miscarriage can predict an abnormal karyotype.

**Methods:**

This retrospective observational cohort study was conducted at the early‐pregnancy assessment unit at University College London Hospital, London, UK, between January 2017 and February 2024. Cytogenetic testing was offered routinely to patients experiencing recurrent miscarriage (at least two miscarriages) or was made available through self‐funding. Eligible participants had a singleton, normally sited pregnancy with evidence of cardiac activity on at least one ultrasound scan, and were subsequently diagnosed with a first‐trimester miscarriage, with successful cytogenetic testing of pregnancy tissue. Crude ultrasound measurements of gestational sac mean diameter (GSMD), yolk sac mean diameter (YSMD), crown–rump length (CRL) and embryonic heart rate from the last live ultrasound scan were converted to centiles according to established biometric reference data. Univariable and multivariable logistic regression analysis was used to assess associations between sonographic features and karyotype result.

**Results:**

Among 158 cases included in the final analysis, 46 (29.1%) had a normal karyotype and 112 (70.9%) had an abnormal karyotype. Those with an abnormal karyotype had a significantly higher median maternal age at conception (38 (interquartile range (IQR), 34–41) years *vs* 35 (IQR, 33–38) years; *P* = 0.0005). Median GSMD centile (*P* = 0.005) and median CRL centile (*P* = 0.003) were lower in pregnancies with an abnormal karyotype, while bradycardia (heart rate < 5^th^ centile) (*P* = 0.04) and enlarged YSMD (≥ 95^th^ centile) (*P* = 0.03) were more common in this group. A combination of four abnormal morphological features (GSMD < 5^th^ centile + YSMD ≥ 95^th^ centile + CRL < 5^th^ centile + bradycardia), termed the ‘tetrad of aneuploidy’, predicted an abnormal karyotype (odds ratio, 7.51 (95% CI, 2.41–140.22); *P* < 0.001), with a specificity of 100% (95% CI, 92.29–100%), for cases last examined sonographically ≤ 10 weeks' gestation.

**Conclusions:**

The presence of a specific combination of abnormal early‐pregnancy ultrasound markers, termed the tetrad of aneuploidy, is a strong predictor of chromosomal abnormality in pregnancies presenting initially with a live embryo. Recognition of this pattern could improve patient counseling and inform clinical decision‐making in early pregnancy. © 2025 The Author(s). *Ultrasound in Obstetrics & Gynecology* published by John Wiley & Sons Ltd on behalf of International Society of Ultrasound in Obstetrics and Gynecology.

## INTRODUCTION

Miscarriage is the most common pregnancy complication, with a pooled risk of 15.3% among all pregnancies^1^. Chromosomal abnormalities are found in approximately 60% of first‐trimester miscarriages^2^, ^3^, the most common being autosomal trisomy, monosomy X and triploidy^3^. In addition to complete aneuploidies, partial trisomy and monosomy resulting from parental balanced translocation have also been reported amongst first‐trimester miscarriages. Understanding the etiopathology of pregnancy loss can often help those affected come to terms with their loss^4^.

The 2018 European Society of Human Reproduction and Embryology (ESHRE) guidelines advised that cytogenetic testing of pregnancy tissue following a miscarriage could be performed for explanatory purposes^4^. Although the diagnosis of a chromosomal abnormality has limited prognostic value for subsequent pregnancy outcome, the finding of a translocation may be associated with recurrent miscarriage if one of the parents carries the same translocation^5^. In addition, androgenic triploidies can be associated with persisting trophoblastic disease that may require specialized oncology follow‐up^6^.

Karyotyping of villous tissue from a miscarriage has several limitations, such as cell‐culture failure, maternal contamination and the high associated cost^7^. High‐resolution transvaginal ultrasonography (TVS) is the primary method for evaluating first‐trimester pregnancies in an early pregnancy assessment unit (EPAU). TVS enables the examination of embryonic structures and the identification of abnormalities by comparison with biometric reference data. Numerous studies have examined the prognostic significance of the presence, size and shape of the gestational sac, yolk sac and embryo, as well as heart rate (HR) patterns, as potential predictors of poor pregnancy outcome^8^, ^9^, ^10^, ^11^, ^12^. A significant increase in the likelihood of chromosomal abnormality was observed when abnormal morphological features were present on TVS^13^. Examples of this include a significant association between an enlarged yolk sac measuring > 95^th^ centile and trisomy 22^13^, ^14^, and between an extreme HR and certain chromosomal abnormalities, such as tachycardia and trisomy 13^9^, ^15^, ^16^.

The aim of this study was to investigate whether combining various abnormal sonographic morphological features of live pregnancies that ended in a first‐trimester miscarriage could be used to differentiate between chromosomally normal and chromosomally abnormal pregnancies.

## METHODS

### Study design and participants

This was a retrospective observational cohort study conducted at a single specialized EPAU at University College London Hospital, London, UK, between January 2017 and February 2024. The EPAU is an open‐access service for pregnant women < 13 + 6 weeks' gestation presenting with pain, bleeding or a history of early‐pregnancy complications.

Eligible study participants were women with a singleton, normally sited (eutopic) pregnancy with evidence of cardiac activity on at least one ultrasound scan, who were subsequently diagnosed with a first‐trimester miscarriage and underwent successful cytogenetic testing of pregnancy tissue. Ultrasound data were collected from the last live pregnancy scan to avoid including degenerative changes in pregnancy morphology that may occur following cessation of embryonic cardiac activity. Patients with a history of irregular menstrual cycles and those with uncertain menstrual or conception dates (such as conception that occurred within three cycles after a previous pregnancy or since stopping contraception) were excluded. No formal ethics approval was required as per the National Health Service Research Ethics Committee and local ethics committee, as the data had already been collected during routine care, were anonymized and were analyzed within the care team.

Maternal characteristics including age, body mass index, ethnicity, mode of conception (spontaneous or assisted), gravidity, parity and medical history were documented and stored in a secure hospital database (Viewpoint; Viewpoint Bildverarbeitung GmbH, Munich, Germany). Gestational age was based on the last menstrual period, date of conception in the case of ovulation induction or date of embryo transfer.

### Ultrasound protocol

All patients underwent a standardized TVS assessment using a high‐resolution 4–9‐MHz transvaginal probe (Voluson E8; GE Healthcare, Zipf, Austria). Examinations were carried out by clinical fellows, all of whom were European Federation of Societies for Ultrasound in Medicine and Biology (EFSUMB) Level‐II operators, under the supervision of specialist gynecologists who were expert Level‐III operators^17^. Standard images were acquired and stored in Viewpoint. Miscarriage diagnosis followed the National Institute of Health and Care Excellence (NICE) guidelines^18^.

On each ultrasound scan, the following early pregnancy structures were examined: (1) gestational sac mean diameter (GSMD) in mm, averaged from three perpendicular diameters with calipers placed at the inner edges of the gestational sac wall^19^; (2) yolk sac mean diameter (YSMD) in mm, averaged from three perpendicular diameters with calipers placed at the center of the yolk sac wall^20^; (3) crown–rump length (CRL) in mm, measured in the sagittal view at its greatest length^21^; (4) presence of an amniotic sac, identified as a spherical structure within the gestational sac, distinct from the yolk sac, in which the embryo is situated^22^; and (5) embryonic HR in bpm, measured by calculating distance between two heart waves on a frozen M‐mode image^23^. Crude measurements of GSMD, YSMD, CRL and HR were converted to centiles using established biometric reference data, namely those of Robinson and Fleming^24^, ^25^ for CRL, Rempen^26^ for GSMD, Grisolia *et al*.^27^ for YSMD and Papaioannou *et al*.^28^ for HR, with HR < 5^th^ centile defined as bradycardia.

### Cytogenetic analysis

Patients diagnosed with a miscarriage were managed expectantly, medically or by surgical evacuation under a general or local anesthetic, according to patient preference. Pregnancy tissue was sent for cytogenetic testing with written consent if patients had experienced two or more miscarriages in previous pregnancies, in line with our routine clinical practice. Women could also elect to self‐fund testing outside this scenario.

All samples were cleaned under the dissecting microscope prior to analysis. Therefore, maternal cell contamination was identified prior to karyotyping and, if found, the case was excluded from the study. Cytogenetic analysis was performed by conventional GTG‐banding, with a laboratory success rate of approximately 70%. For those samples in which cultures failed, a molecular technique, namely the bacterial artificial chromosomes (BACs)‐on‐Beads assay (PerkinElmer, Waltham, MA, USA), was carried out. In addition, quantitative fluorescence polymerase chain reaction was used in cases of suspected triploidy. Cytogenetic analysis of pregnancy tissue was recorded in a binary format (normal/abnormal) to aid analysis and also as a description of the specific chromosomal abnormality detected.

### Statistical analysis

The normality of continuous baseline variables was assessed using the Shapiro–Wilk test. Continuous data are presented as median (interquartile range (IQR)) and categorical data as *n* (%). GSMD, YSMD, CRL and HR centiles are presented in continuous and categorical formats. For the latter, abnormal centile extremes were coded as 1 and values not meeting the preselected criteria were coded as 0. The extreme centile cut‐offs for GSMD, YSMD and CRL were determined by dividing centiles for each morphological feature into 5‐point bins to assess the percentage frequency distribution. From this, we defined cut‐offs associated significantly with abnormal karyotypes applicable to the present cohort. To determine the extreme centile cut‐offs, the following formula was employed: (percentage frequency of abnormal karyotype)/(percentage frequency of normal karyotype). The highest ratio per feature was selected, namely 3.20 for GSMD, 6.03 for YSMD and 2.08 for CRL. This empirical approach established cut‐off values of < 5^th^ centile for GSMD, ≥ 95^th^ centile for YSMD and < 5^th^ centile for CRL (Figure S1).

The cohort was divided into two groups: those with a normal karyotype and those with an abnormal karyotype. To identify statistically significant differences in demographic and ultrasound variables across comparator groups, the Mann–Whitney *U*‐test was used for continuous data and the Fisher's exact test or the chi‐square test, as appropriate, was used for categorical data.

Univariable and multivariable logistic regression analysis was used to investigate the association between sonographic morphological variables, in isolation or in combination, and an abnormal karyotype result. Effect size was expressed as odds ratios (OR) with 95% CIs. Analyses were conducted for those with the last live ultrasound examination performed at any gestational age and for those with the last examination performed ≤ 10 weeks' gestation; 10 weeks was chosen as the cut‐off because embryological research demonstrates yolk‐sac regression after this gestational age^20^. Tjur's R‐squared test was employed to verify non‐linearity. The validity of null hypothesis rejection was assessed using the Hosmer–Lemeshow goodness‐of‐fit test.

Sensitivity, specificity, negative and positive predictive values and accuracy, with 95% CIs, were calculated for GSMD < 5^th^ centile, YSMD ≥ 95^th^ centile, CRL < 5^th^ centile and HR < 5^th^ centile. GSMD, YSMD and CRL, as continuous variables, and bradycardia, as a binary variable, were included as covariates in the multivariable model. A forced‐entry method was used based on the known biological plausibility and clinical relevance of the variables. Multicollinearity was assessed using the variance inflation factor (VIF) and found to be minimal (all VIFs < 2). ORs for abnormal karyotype were calculated using contingency tables (Fisher's exact test with the Koopman asymptotic score) for sonographic morphological features individually and then as a composite ‘tetrad of aneuploidy’ (GSMD < 5^th^ centile + YSMD ≥ 95^th^ centile + CRL < 5^th^ centile + bradycardia).

To complement effect sizes with clinically interpretable test performance (i.e. diagnostic yield of the tetrad of aneuploidy compared with that of each individual sonographic marker), we employed McNemar's test, analyzing discordant pairs in the paired 2 × 2 table (marker positive/tetrad negative *vs* marker negative/tetrad positive). A two‐sided, continuity‐corrected χ^2^ statistic was reported when the number of discordant observations was ≥ 10; for sparser tables, we applied the exact binomial version. For sparse scenarios (e.g. the ‘all‐abnormal’ composite), we summarized the 2 × 2 table and reported the exact Fisher's two‐sided *P*‐value with a continuity‐corrected OR. A prespecified subgroup analysis was performed for assessment ≤ 10 weeks' gestation using the same framework. We calculated diagnostic accuracy estimates for each predictor and the composite where possible, and, for congruence, presented them alongside adjusted ORs (95% CI) and *P*‐values from the multivariable model.

All statistical tests were two‐sided, with the significance threshold set at *P* ≤ 0.05. Statistical analysis and graph generation were performed using GraphPad version 10 (GraphPad Software, LLC., Boston, MA, USA).

## RESULTS

### Demographic characteristics

**Figure 1:**
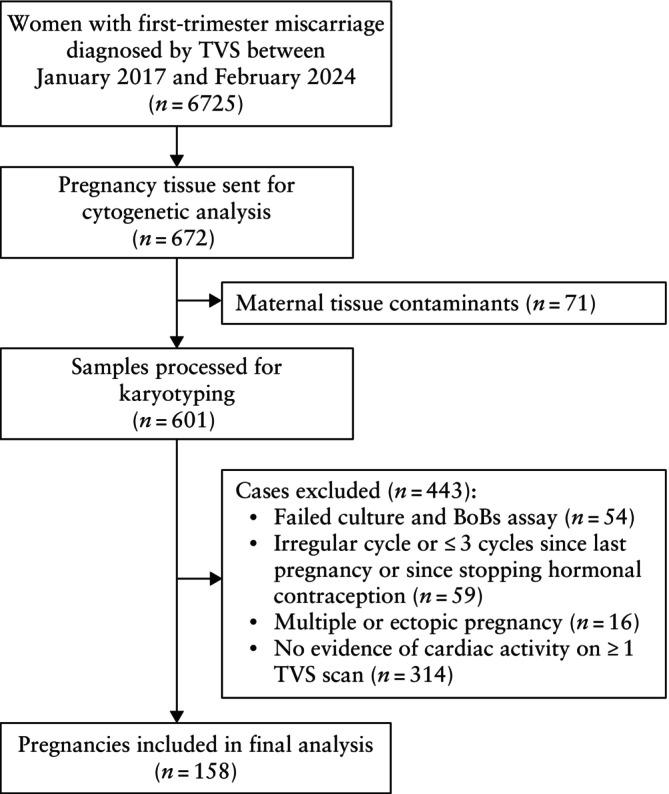
Flowchart summarizing inclusion of women attending early‐pregnancy unit during study period. BoBs assay, bacterial artificial chromosomes (BACs)‐on‐Beads assay; TVS, transvaginal ultrasound.

Of 6725 first‐trimester miscarriages diagnosed between January 2017 and February 2024, 158 cases met the inclusion criteria (Figure 1). Of those, 22 (13.9%) patients underwent self‐funded cytogenetic testing, while 136 (86.1%) patients received routine testing owing to a history of two or more miscarriages.

Cytogenetic analysis revealed 46 (29.1%) normal and 112 (70.9%) abnormal karyotype results (Table 1). There was a significantly higher median maternal age at conception in the group with an abnormal karyotype (38 (IQR, 34–41) years *vs* 35 (IQR, 33–38) years; *P* = 0.0005), while other demographic and clinical characteristics did not differ significantly between the groups. The median gestational age at miscarriage in the overall cohort was 67 (IQR, 60–72) days, with no significant difference between the two groups.

**Table 1:** Baseline characteristics of 158 pregnancies that underwent first‐trimester miscarriage, according to karyotype result

Characteristic	Normal karyotype (*n* = 46)	Abnormal karyotype (*n* = 112)	*P* *
Maternal age at conception (years)	35 (33–38)	38 (34–41)	0.0005
Body mass index† (kg/m^2^)	23.4 (20.9–26.2)	24.5 (22.0–29.0)	0.98
Self‐reported ethnicity			0.64
Caucasian	23 (50.0)	65/111 (58.6)	
Mixed/Other	15 (32.6)	25/111 (22.5)	
Afro‐Caribbean	3 (6.5)	8/111 (7.2)	
East Asian	3 (6.5)	8/111 (7.2)	
South Asian	2 (4.3)	5/111 (4.5)	
Gravidity	3 (2–5)	4 (2–5)	0.12
Parity	0 (0–1)	0 (0–1)	0.99
≥ 3 prior miscarriages	28 (60.9)	67 (59.8)	0.13
Mode of conception			0.26
Spontaneous	38 (82.6)	93 (83.0)	
ART	8 (17.4)	19 (17.0)	
GA at miscarriage (days)	61 (58–72)	68 (62–73)	0.11

Data are given as median (interquartile range), *n* (%) or *n*/*N* (%).

* Continuous variables were compared using Mann–Whitney *U*‐test and categorical variables were compared using chi‐square test or Fisher's exact test, as appropriate.

† Data missing for 40 patients. ART, assisted reproductive technology; GA, gestational age.

**Figure 2:**
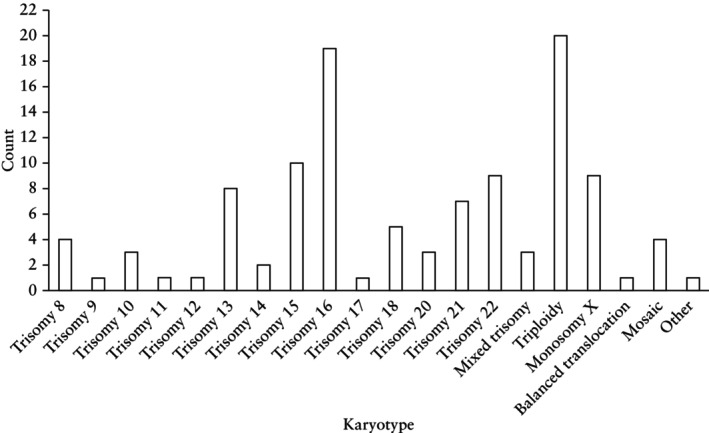
Distribution of abnormal karyotype results in 112 cases of first‐trimester miscarriage.

Among those with an abnormal karyotype, autosomal trisomies were the most common chromosomal abnormality (77/112 (68.8%)), followed by triploidy (20/112 (17.9%)) and monosomy X (9/112 (8.0%)) (Figure 2). Trisomy 16 was the most frequent trisomy (19/77 (24.7%)), followed by trisomy 15 (10/77 (13.0%)) and trisomy 22 (9/77 (11.7%)).

### Relationships between ultrasound and cytogenetic findings

Table 2 shows the measurements of GSMD, YSMD, CRL and HR and the frequency of amniotic sac presence in conceptuses with a normal karyotype and those with an abnormal karyotype. The median GSMD and CRL centiles were significantly lower in those with an abnormal karyotype, but there was no significant difference between the groups in the rate of extreme measurements, i.e. < 5^th^ centile or > 95^th^ centile. Bradycardia (HR < 5^th^ centile) occurred more frequently in those with an abnormal karyotype (*P* = 0.04), as did an abnormally enlarged YSMD ≥ 95^th^ centile (*P* = 0.03). The presence of an amniotic sac was analyzed in all 78 pregnancies progressing beyond 7 + 0 weeks; no significant difference was noted between the two groups.

**Table 2:** Sonographic morphological features at last live ultrasound scan (at any gestational age) before first‐trimester miscarriage, according to karyotype result

Sonographic feature	Normal karyotype (*n* = 46)	Abnormal karyotype (*n* = 112)	*P* *
GSMD centile	28 (11–50)	13 (3–32)	0.005
GSMD (categorical)			0.09
> 95^th^ centile	2 (4.3)	2 (1.8)	
< 5^th^ centile	5 (10.9)	42 (37.5)	
YSMD centile	14 (3–39)	26 (6–70)	0.06
YSMD (categorical)			0.03
≥ 95^th^ centile	2 (4.3)	19 (17.0)	
< 5^th^ centile	14 (30.4)	23 (20.5)	
CRL centile	9 (3–38)	3 (3–27)	0.003
CRL (categorical)			0.06
> 95^th^ centile	1 (2.2)	0 (0)	
< 5^th^ centile	11 (23.9)	71 (63.4)	
Heart rate (bpm)	119.5 (89.8–159.0)	113.0 (88.3–148.8)	0.77
Heart rate (categorical)			0.04
Normal†	26 (56.5)	45 (40.2)	
Bradycardia‡	16 (34.8)	61 (54.5)	
Amniotic sac present§			0.09
No	6/19 (31.6)	33/59 (55.9)	
Yes	13/19 (68.4)	26/59 (44.1)	

Data are given as median (interquartile range), *n* (%) or *n*/*N* (%).

* Continuous variables were compared using Mann–Whitney *U*‐test and categorical variables were compared using chi‐square test or Fisher's exact test, as appropriate.

† Defined as heart rate between 5^th^ and 95^th^ centiles.

‡ Defined as heart rate < 5^th^ centile.

§ Includes only cases progressing beyond a gestational age of 49 days. CRL, crown–rump length; GSMD, gestational sac mean diameter; YSMD, yolk sac mean diameter.

Multiple logistic regression analysis confirmed that a smaller gestational sac (OR, 0.98 (95% CI, 0.96–0.99); *P* = 0.03) and an increasing YSMD (OR, 1.02 (95% CI, 1.00–1.03); *P* = 0.03) were associated significantly with an abnormal karyotype, both in pregnancies evaluated at any gestational age and in the subgroup evaluated ≤ 10 weeks (Table S1). Four‐way interaction logistic regression analysis of the sonographic morphological features highlighted a positive association with abnormal karyotype in pregnancies evaluated ≤ 10 weeks (OR, 2.94 (95% CI, 2.01–4.38); *P* < 0.0001), suggesting that the presence of all the aforementioned sonographic features may successfully predict karyotype status in pregnancies ≤ 10 weeks.

**Figure 3:**
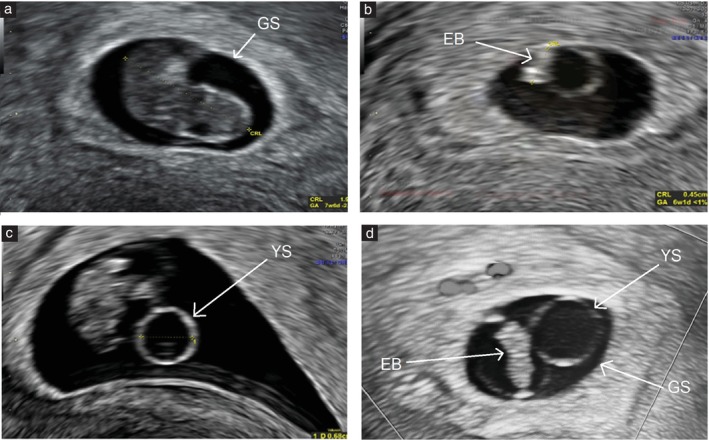
Two‐dimensional transvaginal ultrasound images of abnormal pregnancy morphological features shown individually (a–c) and in combination (d). (a) Small gestational sac (GS) measuring < 5^th^ centile. (b) Embryo (EB) with short crown–rump length (CRL) (calipers) measuring < 5^th^ centile. (c) Enlarged yolk sac (YS) measuring ≥ 95^th^ centile (calipers). (d) Case with combination of abnormal features: GS mean diameter < 5^th^ centile + YS mean diameter ≥ 95^th^ centile + CRL < 5^th^ centile. GA, gestational age.

The odds of an abnormal karyotype were evaluated according to the presence of the predefined sonographic morphological abnormalities, both individually and in combination (Table 3). When assessed individually, each abnormal ultrasound feature was associated significantly with an abnormal karyotype across all gestational ages, with CRL < 5^th^ centile demonstrating the highest OR (5.51 (95% CI, 2.53–12.01); *P* < 0.001). In combination, the tetrad of aneuploidy (GSMD < 5^th^ centile + YSMD ≥ 95^th^ centile + CRL < 5^th^ centile + bradycardia) was associated significantly with abnormal karyotypes specifically in cases examined ≤ 10 weeks (OR, 7.51 (95% CI, 2.41–140.22); *P* < 0.001). Ultrasound images of abnormal morphological findings on TVS are provided in Figure 3.

**Table 3:** Univariable logistic regression analysis of association of individual and combined sonographic morphological features with abnormal karyotype result, according to gestational age (GA) at last live ultrasound scan before first‐trimester miscarriage

Sonographic feature	OR (95% CI)	*P*
All GA (*n* = 158)		
GSMD < 5^th^ centile	4.92 (1.80–13.43)	0.001
YSMD ≥ 95^th^ centile	4.50 (1.00–20.15)	0.04
CRL < 5^th^ centile	5.51 (2.53–12.01)	< 0.001
Bradycardia*	2.24 (1.10–4.57)	0.03
Tetrad of aneuploidy†	8.54 (0.49–149.77)	0.06
GA ≤ 10 weeks (*n* = 61)		
GSMD < 5^th^ centile	8.57 (1.76–41.78)	0.004
YSMD ≥ 95^th^ centile	4.61 (0.53–39.71)	0.25
CRL < 5^th^ centile	2.63 (0.88–7.90)	0.09
Bradycardia*	2.12 (0.71–6.28)	0.27
Tetrad of aneuploidy†	7.51 (2.41–140.22)	< 0.001

* Defined as heart rate < 5^th^ centile.

† Defined as gestational sac mean diameter (GSMD) < 5^th^ centile + yolk sac mean diameter (YSMD) ≥ 95^th^ centile + crown–rump length (CRL) < 5^th^ centile + bradycardia. OR, odds ratio.

The tetrad of aneuploidy had a specificity of 100% (95% CI, 92.29–100%) for abnormal karyotype, with a positive predictive value of 100% (95% CI, 66.37–100%), although sensitivity was low at 23.54% (95% CI, 17.82–29.88%) (Table 4). Nine cases exhibited the tetrad of aneuploidy, all of which had an abnormal karyotype: eight cases of autosomal trisomy (six of trisomy 16, one of trisomy 10 and one of trisomy 10 with multiple deletions) and one case of triploidy. All genetic abnormalities were lethal; none could have potentially resulted in a live birth.

**Table 4:** Diagnostic accuracy of individual and combined sonographic morphological features for predicting abnormal karyotype result ≤ 10 weeks' gestation (*n* = 61)

Sonographic feature	Sensitivity (%) (95% CI)	χ^2^ (1 df)*	*P*	Specificity (%) (95% CI)	χ^2^ (1 df)*	*P*	NPV (%) (95% CI)	PPV (%) (95% CI)	Accuracy (%) (95% CI)	aOR (95% CI)†	*P*
GSMD < 5^th^ centile	19.64 (13.34–27.95)	31.0	< 0.0001	93.48 (82.50–97.76)	3.2	0.07	58.56 (54.17–62.82)	76.64 (58.12–88.57)	62.95 (54.81–70.59)	1.06 (0.40–2.83)	0.902
YSMD ≥ 95^th^ centile	7.14 (3.66–13.46)	8.1	0.004	97.83 (88.66–99.89)	0.5	0.48	54.29 (51.76–56.81)	87.76 (49.80–98.11)	57.71 (49.27–65.84)	3.28 (1.20–8.95)	0.021
CRL < 5^th^ centile	28.57 (21.02–0.37)	60.0	< 0.0001	76.09 (62.06–86.09)	9.1	0.003	61.90 (53.42–69.72)	60.87 (51.49–69.51)	61.36 (53.19–69.09)	3.61 (0.77–16.94)	0.104
Bradycardia‡	26.79 (19.45–35.66)	50.0	< 0.0001	78.26 (64.43–87.74)	14.1	< 0.001	58.82 (51.28–65.98)	60.40 (49.92–70.00)	59.55 (51.35–67.37)	1.67 (0.64–4.32)	0.292
Tetrad of aneuploidy§	23.54 (17.82–29.88)	—	—	100 (92.29–100)	—	—	52.13 (50.74–53.52)	100 (66.37–100)	54.09 (45.88–62.14)	NC	NC

* McNemar χ^2^ test comparing each individual marker with tetrad of aneuploidy, where χ^2^ statistic reflects two‐sided, continuity‐corrected calculation with 1 degree of freedom (df).

† Adjusted for other three sonographic features in multivariable model.

‡ Defined as heart rate < 5^th^ centile.

§ Defined as gestational sac mean diameter (GSMD) < 5^th^ centile + yolk sac mean diameter (YSMD) ≥ 95^th^ centile + crown–rump length (CRL) < 5^th^ centile + bradycardia. aOR, adjusted odds ratio; NC, non‐calculable; NPV, negative predictive value; PPV, positive predictive value.

## DISCUSSION

### Main findings

This study is the first to demonstrate that a combination of four abnormal morphological first‐trimester ultrasound features (GSMD < 5^th^ centile, YSMD ≥ 95^th^ centile, CRL < 5^th^ centile and bradycardia) can predict an abnormal karyotype with excellent specificity, a finding that has the potential to improve the counseling and management of patients presenting with suspected complications in early pregnancy. While previous studies have noted associations between individual features and chromosomal abnormalities^8^, ^13^, ^19^, ^29^, ^30^, ^31^, ^32^, ^33^, ^34^, none explored the value of combining multiple markers to enhance diagnostic performance.

### Comparison with other studies

In our cohort, 70.9% of first‐trimester miscarriages had an abnormal karyotype, concordant with previously reported rates of 61.4–66%^9^, ^13^, ^29^, ^32^. Autosomal trisomies, particularly trisomy 16, were the most common abnormalities in our cohort, consistent with previous evidence^35^. A previous review reported a 17% prevalence of polyploidy in first‐trimester miscarriages^35^, comparable with our findings. Similar to other studies, maternal age at conception was significantly higher in the group with an abnormal karyotype^13^, ^15^. This alignment with existing data supports the generalizability of our findings to broader populations.

Isolated gestational‐sac abnormalities, specifically arrested symmetrical growth (GSMD < 5^th^ centile and CRL < 5^th^ centile) and ‘small gestational sac syndrome’ (GSMD < 5^th^ centile and normal CRL) have been linked previously with chromosomal abnormalities^13^, ^33^. Similarly, in our study, isolated GSMD < 5^th^ centile was associated significantly with abnormal karyotypes (OR, 4.92; *P* = 0.001) across all gestational ages.

The yolk sac plays a key role in maternofetal transport, and significant changes in size may reflect dysfunction at this interface, accounting for poor pregnancy outcomes^36^. Definitions of an enlarged yolk sac vary, including YSMD > 95^th^ centile^13^, > 5 mm^29^ or > 6 mm^31^. Although previous studies have found significant associations between isolated enlarged YSMD and aneuploidy, specifically trisomy 22^16^, ^30^, our study showed that YSMD ≥ 95^th^ centile measured ≤ 10 weeks had a specificity of 98% for an abnormal karyotype, but its OR did not reach significance (*P* = 0.25).

In the study of Dickey *et al*.^8^, CRL < 50^th^ centile after day 28 postovulation was associated with an increased risk of miscarriage. While early growth appears normal in some aneuploid embryos (trisomy 15 and trisomy 21), trisomy 16 has been associated with trophoblastic hypoproliferation and early fetal growth restriction^37^. In our cohort, conceptuses with an abnormal karyotype had a significantly smaller median CRL (3^rd^ centile *vs* 9^th^ centile; *P* = 0.003). A CRL < 5^th^ centile across all gestational ages was associated significantly with abnormal karyotypes (OR, 5.51; *P* < 0.001).

Bradycardia or tachycardia in first‐trimester pregnancies is most probably due to anomalies of embryonic cardiovascular development, and can be linked to underlying chromosomal abnormalities^9^, ^15^, ^16^ or imbalances in the autonomic nervous system^38^. Embryonic bradycardia has been linked to severe early‐onset fetal growth restriction associated with trisomy 18 and triploidy^39^, ^40^. In these cases, bradycardia is a preterminal event^16^, rather than a result of autonomic nervous system disruption, as parasympathetic development does not begin until 11 weeks' gestation^38^. By contrast, tachycardia has been associated with trisomy 13 and Turner syndrome, in which narrowing of the left ventricle outflow tract activates aortic arch baroreceptors, triggering a tachycardic response^16^. In the present study, isolated bradycardia was associated with an increased risk of abnormal karyotype (OR, 2.24; *P* = 0.03) across all gestational ages.

Although the 95% CI of the OR for the tetrad of aneuploidy overlapped with those of individual sonographic markers, reflecting the tetrad's rarity and wider 95% CI, the tetrad uniquely achieved 100% specificity and positive predictive value, thresholds not reached by any individual marker. The simultaneous presence of multiple embryonic abnormalities enhances biological plausibility for aneuploidy compared with isolated abnormalities, which may reflect measurement variability or physiological deviations. A significant four‐way interaction in our multiple logistic regression analysis further supports a synergistic effect, reinforcing the tetrad's diagnostic potential in early pregnancy.

### Strengths and limitations

A key strength of this study is our inclusion of only cases with evidence of cardiac activity prior to miscarriage, ensuring that ultrasound measurements of embryonic structures were not influenced by postmortem tissue degradation. Additionally, restricting inclusion to cases with a known conception date or regular menstrual cycles allowed for accurate centile measurements based on biometric reference data. Homogeneity in the quality of ultrasound measurements was maintained, with all scans performed by highly experienced operators. Furthermore, multivariable analysis, applied rarely in similar studies, and the combination of multiple abnormal markers enhance the robustness of our findings.

Limitations of the study include the retrospective design and selection bias, as only women meeting local criteria for karyotyping or those with the resources to self‐fund cytogenetic testing were included. In addition, our sample size was relatively small and there was a significant proportion of culture failures. Although the 2021 *Lancet* series on recurrent miscarriages recommended a graded approach to miscarriage management^41^, routine karyotyping remains unavailable for most single sporadic first‐trimester losses. As a result, most patients do not undergo karyotyping and, when attempted, culture failure is common, which may leave the cause of miscarriage unexplained and compound psychological distress. This study aimed to assess whether specific combinations of ultrasound features could reliably indicate chromosomal abnormalities in first‐trimester miscarriages. Such an approach may be particularly valuable in cases for which karyotyping is not indicated or is unsuccessful, providing additional information regarding the probable etiology of pregnancy loss.

### Future perspectives

EPAU care has a positive overall effect on women's health and emotional wellbeing^42^. Ultrasound‐based counseling on the likely outcome of pregnancy may help to alleviate anxiety associated with diagnostic uncertainty. The presence of the tetrad of aneuploidy could assist counseling by indicating that, despite the presence of embryonic cardiac activity, the features are predictive of an abnormal karyotype and a follow‐up scan is necessary to determine pregnancy outcome.

If validated in larger prospective studies, the 100% specificity of the tetrad of aneuploidy for abnormal karyotype could support a more personalized approach to the management of miscarriage. Future research should consist of larger, prospective and consecutive cohorts with propensity‐score matching to reduce bias between groups, increasing the prediction capacity of miscarriage secondary to karyotype abnormality. This would allow for more targeted testing, reducing the cost associated with cytogenetic testing. Early detection of chromosomal abnormalities would enable timely karyotyping, and in cases of balanced translocations, facilitate prompt parental testing and genetic counseling.

### Conclusions

This study presents novel evidence that a specific combination of abnormal first‐trimester ultrasound markers, termed the tetrad of aneuploidy, could aid in detecting chromosomal abnormalities and stratifying miscarriage risk in pregnancies with an initially live embryo. These results suggest a role for ultrasound in providing diagnostic insights beyond viability assessment, aiding in the counseling and management of patients at risk of early pregnancy complications. Validation in large‐scale prospective cohorts with consecutive recruitment and propensity‐matched controls is needed to confirm the diagnostic accuracy of this sonographic pattern.

## Supporting information


**Figure S1** Distribution of normal (blue) and abnormal (red) karyotype results per 5‐centile category of gestational sac mean diameter (GSMD) (a), yolk sac mean diameter (YSMD) (b) and crown–rump length (CRL) (c). Centile cut‐offs for cases at ≤ 10 weeks' gestation (70 days) are indicated by dashed lines.

**Table S1** Multiple logistic regression analysis of association between sonographic morphological features and abnormal karyotype result.

## Data Availability

The data that support the findings of this study are available from the corresponding author upon reasonable request.
