# Supplementary figures and images for: Ultrasound features in early pregnancy for predicting abnormal karyotype in first‐trimester miscarriage
Source: Ultrasound Obstet Gynecol. 2026 Jan 3;67(3):376–84. doi: 10.1002/uog.70159 (PMC12951263; doi:10.1002/uog.70159)

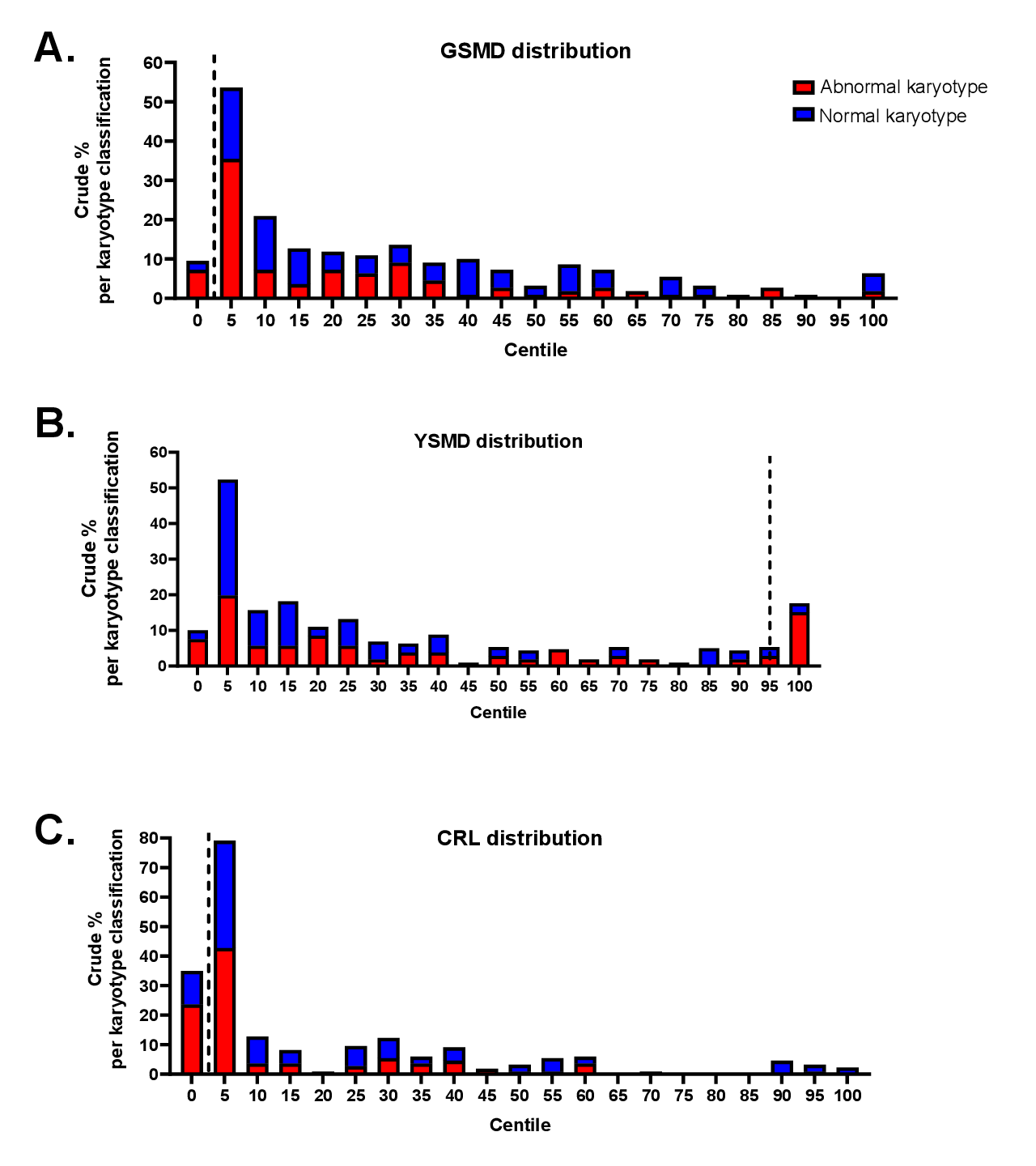

Supplement: Supplementary file 1 — Figure S1 Distribution of normal (blue) and abnormal (red) karyotype results per 5‐centile category of gestational sac mean diameter (GSMD) (a), yolk sac mean diameter (YSMD) (b) and crown–rump length (CRL) (c). Centile cut‐offs for cases at ≤ 10 weeks' gestation (70 days) are indicated by dashed lines. [file UOG-67-376-s001.tif]
