# Supplementary material for: Ultrasound features in early pregnancy for predicting abnormal karyotype in first‐trimester miscarriage
Source: Ultrasound Obstet Gynecol. 2026 Jan 3;67(3):376–84. doi: 10.1002/uog.70159 (PMC12951263; doi:10.1002/uog.70159)
Supplement: Supplementary file 2 — Table S1 Multiple logistic regression analysis of association between sonographic morphological features and abnormal karyotype result. [file UOG-67-376-s002.docx]

**Table S1** Multiple logistic regression analysis of association between sonographic morphological features and abnormal karyotype result

| **Gestational age of ultrasound examination** | **Ultrasound morphological features** | **Odds ratio** | **95% CI** | **\|Z\|** | **P value** |
| --- | --- | --- | --- | --- | --- |
| **All GA** | GSMD | 0.98 | 0.96 - 0.99 | 2.05 | 0.03 |
|  | YSMD | 1.02 | 1.00 –1.03 | 2.08 | 0.03 |
|  | CRL | 0.99 | 0.97 - 1.01 | 0.28 | 0.16 |
|  | Bradycardia | 1.74 | 0.71 - 4.31 | 1.21 | 0.34 |
|  |  | | | | |
|  | Four-way interaction GSMD, YSMD, CRL and bradycardia | **1.09** | **0.37 - 3.27** | **0.17** | 0.27 |
| **GA ≤10 weeks gestation** | GSMD | 0.98 | 0.96 - 0.99 | 2.03 | 0.04 |
|  | YSMD | 1.02 | 1.003 - 1.03 | 2.24 | 0.02 |
|  | CRL | 1.004 | 0.98 - 1.02 | 0.35 | 0.76 |
|  | Bradycardia | 1.79 | 0.72 - 4.51 | 1.26 | 0.64 |
|  |  | | | | |
|  | Four-way interaction GSMD YSMD, CRL and bradycardia | **2.94** | **2.01 - 4.38** | **5.46** | **<0.0001** |

CRL, crown rump length; GSMD, gestational sac mean diameter; YSMD, yolk sac mean diameter
